# Adverse events associated with basivertebral nerve radiofrequency ablation: A comprehensive analysis from the MAUDE database

**DOI:** 10.1016/j.inpm.2026.100801

**Published:** 2026-07-09

**Authors:** Azadeh Fischer, Amanda N. Cooper, Flynn McGuire, Reza Ehsanian, Allison Glinka Pryzbysz, Zachary L. McCormick, Alexandra E. Fogarty

**Affiliations:** a Department of Physical Medicine and Rehabilitation, New York Medical College, New York, NY, United States; b Department of Physical Medicine and Rehabilitation, University of Utah School of Medicine, Salt Lake City, UT, United States; c Division of Pain Medicine, Department of Anesthesiology and Critical Care Medicine, University of New Mexico School of Medicine, Albuquerque, NM, United States

**Keywords:** Adverse events, Basivertebral nerve ablation, MAUDE

## Abstract

**Background:**

Basivertebral nerve ablation (BVNA) is a minimally invasive intraosseous radiofrequency procedure for vertebrogenic chronic low back pain (CLBP). Although randomized controlled trials have established its efficacy, real-world safety data remain limited.

**Purpose:**

Post-market adverse event (AE) characterization associated with BVNA using the U.S. Food and Drug Administration (FDA) Manufacturer and User Facility Device Experience (MAUDE) database.

**Study design:**

Retrospective analysis of a national medical device adverse event database.

**Methods:**

The MAUDE database was queried for all reports involving FDA-cleared BVNA systems from inception to 2025. Reports were manually reviewed, deduplicated, and coded for AE type, severity, timing, and outcome. Frequencies were summarized with descriptive statistics.

**Results:**

A total of 96 unique medical device reports were analyzed. Vertebral compression fracture was the most frequently reported AE, occurring in 21.9% (21/96) of reports. Among the 21 VCF-containing reports, timing was documented in 12 (57.1%): 6 occurred within 30 days post-procedure (range: 4-21 days) and 6 occurred beyond 30 days (range: 33-294 days). Timing was not reported in the remaining 9 reports. An additional 2 reports described posterior element fractures and are reported separately (2.1%, 2/96). Other common AEs included increased low back pain (10.4%), superficial bleeding (2.1%), and emergency department visits (7.3%). Severe AEs were infrequent, including hospitalization (9.4%), retroperitoneal hematoma (7.3%), and death (2.1%). Both deaths were associated with anesthesia-related events rather than the BVNA device or procedure. No cases of infection, spinal/epidural hematoma, or major neurologic injury were identified. Most reports were manufacturer-submitted, with few voluntary reports or device returns.

**Conclusions:**

This MAUDE-based review complements clinical trial data by characterizing the distribution of post-market adverse event reports associated with BVNA. Vertebral fracture was the most frequently reported event, with variable timing ranging from the day of the procedure to nearly 300 days post-BVNA; no consistent temporal pattern could be established. These findings do not permit conclusions about incidence or causality but identify vertebral fracture and pain as the most represented post-market signals, and highlight the need for prospective registries with standardized follow-up, pre-procedural bone health documentation, and detailed AE capture to more precisely define the long-term safety profile of BVNA.

## 1 Introduction

Chronic low back pain (CLBP) remains a leading cause of disability worldwide and is increasingly recognized as a heterogeneous disorder with multiple potential pain generators. Among these, a distinct vertebrogenic etiology has emerged, characterized by nociceptive signaling from the vertebral endplates via the basivertebral nerve. Structural changes at the endplate—identified on MRI as Modic type 1 or type 2 changes—serve as radiographic biomarkers for candidates of basivertebral nerve ablation (BVNA), a targeted intervention that interrupts these nociceptive pathways [1,2].

BVNA, an intraosseous radiofrequency ablation technique, has been evaluated in several high-quality randomized controlled trials (RCTs) and prospective cohort studies. Sham-controlled and multicenter RCTs have demonstrated significant and durable reductions in pain and disability through 2- and 5-year follow-up periods [[3], [4], [5]]. Long-term pooled analyses corroborate these findings, showing sustained improvements in Oswestry Disability Index (ODI) and pain intensity at 5 years [6]. Furthermore, single-arm and real-world cohort studies have reinforced both the generalizability and safety of BVNA outside controlled research settings [3,7].

Beyond clinical outcomes, BVNA has been associated with reductions in healthcare utilization and cost burden. Long-term pooled data demonstrate decreases in opioid consumption, spinal injections, and surgical interventions relative to baseline, alongside lower rates of lumbar fusion compared with non-surgically managed populations [8]. Economic evaluations similarly indicate that BVNA achieves favorable cost-effectiveness compared with continued conservative management [8,9].

Despite a robust body of evidence supporting its efficacy and value, real-world safety data on procedure-related complications remain relatively sparse. Most published safety findings are derived from RCTs and small prospective series that, while rigorously conducted, are often underpowered to capture rare but clinically significant adverse events. Large-scale, real-world evaluations of BVNA safety—particularly those leveraging post-market surveillance data—are notably lacking.

Accordingly, the present study provides the first analysis of adverse events associated with BVNA using the U.S. Food and Drug Administration's Manufacturer and User Facility Device Experience (MAUDE) database. By describing adverse event reports associated with BVNA within the broader context of interventional spine procedures, this study aims to highlight areas for further investigation, support informed patient counseling, and identify priorities for future safety monitoring and device innovation.

## 2 Methods

### 2.1 Data source

We queried the U.S. Food and Drug Administration (FDA) Manufacturer and User Facility Device Experience (MAUDE) database to identify medical device reports (MDRs) involving basivertebral nerve ablation (BVNA) systems used for vertebrogenic low back pain. The MAUDE database contains adverse event reports submitted by manufacturers, importers, device user facilities, and voluntary reporters. All searches and downloads were performed on August 20, 2025 using the FDA's public online search interface [10].

### 2.2 Device selection

The search strategy targeted all BVNA systems with FDA 510(k) clearance under product code QAI (“Ablation, radiofrequency, intraosseous, for pain relief”) and associated consumables (introducer cannulae, curved/straight probes, and cooled radiofrequency electrodes) that are marketed in the United States for BVNA. The two FDA approved systems included are Intracept® Intraosseous Nerve Ablation System (formerly Relievant Medsystems, now Boston Scientific; FDA approved 2016) and OptaBlate® BVN Basivertebral Nerve Ablation System (Stryker; FDA approved 2025).

### 2.3 Search strategy

Search queries combined the verified 510(k) numbers with relevant brand and model names (e.g., “Intracept,” “Relievant”) to capture reports where the device may have been recorded under alternate labeling. Searches were run individually for each device/system and then merged to form the aggregate dataset. Devices known only from international CE-mark registrations without evidence of U.S. marketing were excluded.

### 2.4 Data extraction and cleaning

All retrieved MDRs were exported in.csv format and processed to remove duplicate entries, manufacturer names were normalized, and reports to device categories were assigned. Two authors independently reviewed the processed data. Variables including year, reporter type, source of MDR submission, device return status, anatomic region, adverse event category, and free-text narrative were reported where possible.

Narratives were manually reviewed to confirm procedural relevance. Some reports included multiple adverse events; in these cases, each distinct AE episode was separately coded and categorized. AE categories included procedure-related complications (vertebral compression fracture, increased low back pain, superficial bleeding, increased radicular pain, and lower extremity weakness), emergency room visits, and severe AEs (SAEs). SAEs included potentially life-threatening complications (e.g., retroperitoneal hematoma), as well as any instances of hospitalization or death. Fractures were classified anatomically based on narrative descriptions as vertebral body compression fractures, involving the anterior or middle column of the vertebral body, or posterior element fractures, involving the lamina, pedicle, transverse process, or pars interarticularis. Reports describing posterior element fractures were categorized separately from vertebral compression fractures.

### 2.5 Data analysis

Frequencies and percentages were calculated for each adverse event category, with results presented as both proportions and raw counts (n/N). Percentages represent the proportion of unique medical device reports (MDRs) containing a given event rather than true incidence estimates, given the inherent limitations of passive surveillance data in the MAUDE database. Descriptive analyses were used to summarize adverse event types, severity, timing, and outcomes.

Descriptive statistics were used throughout. Given the passive surveillance nature of the dataset and the absence of a denominator for total BVNA procedures performed, no inferential statistical comparisons were performed between AE subgroups.

## 3 Results

### 3.1 Dataset and report characteristics

Our analysis was based on a curated working dataset of 201 medical device reports (MDRs) related to basivertebral nerve ablation (BVNA). After removal of duplicate entries—first by identical narrative text and then by consensus review of clinically duplicative reports—96 unique MDRs submitted to the FDA between November 15, 2018 and July 1, 2025 remained for analysis. Each MDR contained a free-text narrative and accompanying structured metadata. All narratives were manually reviewed to confirm procedural relevance and to ensure that reports reflected true BVNA-related adverse events.

### 3.2 Adverse events

Across the 96 unique MDRs, multiple adverse events (AEs) could occur within a single report, producing more AE episodes than total MDRs. Relative frequencies of AEs and severe AEs across the 96 MDRs are summarized by category in Table 1a, Table 1b. The following frequencies reflect the proportion of MDRs in which each event was documented and should not be interpreted as estimates of true clinical incidence, as reporting rates likely vary substantially across event types and the total number of BVNA procedures performed is unknown. The most frequently identified AE was vertebral compression fracture, described in 21 (21.9%, 21/96) MDRs. Two additional MDRs described posterior element fractures (lamina, transverse process, or pars fractures), which are reported separately (2.1%, 2/96). Other reported AEs included increased low back pain in 10 (10.4%, 10/96) MDRs; this category was derived from free-text narrative descriptions and reflects a non-standardized, reporter-defined characterization without defined duration, severity threshold, or temporal criteria. Superficial bleeding was described in 2 (2.1%, 2/96) MDRs, and visit to the emergency room (ER) in 7 (7.3%, 7/96) MDRs. Additionally identified AEs included increased radicular pain in 3 (3.1%, 3/96) MDRs and lower extremity weakness in 1 (1.0%, 1/96) MDR. No MDRs described spinal or epidural hematoma, infection, serious neurologic injury, or deep vein thrombosis/clot.

**Table 1a:** Frequency of adverse events described in 96 medical device reports related to basivertebral nerve ablation.

Adverse Event	n/N (%)
Vertebral compression fracture	23/96 (24.0)
Increased low back pain	10/96 (10.4)
Superficial bleeding	2/96 (2.1)
Visit to the emergency room	7/96 (7.3)
Increased radicular pain	3/96 (3.1)
Lower extremity weakness	1/96 (1.0)

**Table 1b:** Frequency of severe adverse events described in 96 medical device reports related to basivertebral nerve ablation.

Severe Adverse Event	n/N (%)
Hospitalization	9/96 (9.4)
Retroperitoneal hematoma	7/96 (7.3)
Death	2/96 (2.1)

Severe adverse events were also identified in the dataset. Hospitalization occurred in 9 (9.4%, 9/96) MDRs, retroperitoneal hematoma in 7 (7.3%, 7/96) MDRs, and death in 2 (2.1%, 2/96) MDRs. Both deaths were associated with anesthesia-related events rather than the BVNA device or procedure. In one case, the procedure was completed successfully and the patient subsequently experienced post-procedural anesthesia complications, with the treating physician explicitly noting the death was unrelated to the device or procedure. In the second case, an intraoperative cardiorespiratory event occurred including BP drop, cardiac arrest, and airway obstruction, consistent with an anesthesia-related complication, though this attribution was not explicitly confirmed in the narrative. Neither report specified the type of anesthesia administered. Severe events often co-occurred (e.g., retroperitoneal hematoma with hospitalization and/or ER visit). Level specification varied by event type. Among fracture-containing MDRs, the vertebral level was documented in the majority of reports and consistently involved the lumbar spine. In contrast, level was infrequently documented in anesthesia-related and device malfunction reports, for which vertebral level is less clinically relevant. Overall, 44 of 96 MDRs (45.8%) specified at least one vertebral level, all within the lumbar spine (L1-S1). Timing information was inconsistently reported. For non-fracture events including anesthesia complications, bleeding, and device malfunctions, timing was most often described as intraoperative or occurring on the day of the procedure. For fracture-containing MDRs, timing is described separately below. The most common initial reporter occupation was physician (39.6%, 38/96), followed by non-healthcare professional (24.0%, 23/96) and other (18.8%, 18/96). These two fields are not contradictory: the initial reporter identifies who first observed or communicated the event, while the submission source reflects who formally filed the MDR with the FDA. In most cases, a physician reported the event to the manufacturer, who then submitted the formal MDR. Nearly all MDRs (86.5%, 83/96) were submitted by manufacturers rather than user facilities (7.3%, 7/96) or voluntary reporters (6.3%, 6/96), and only a minority (22.9%, 22/96) of devices were returned for evaluation. Reported outcomes ranged from conservative management to hospitalization or subsequent spine surgery (fusion, decompression, or kyphoplasty).

#### 3.2.1 Vertebral compression fractures and timing

Vertebral compression fractures were the most frequently identified AE in this dataset but were infrequently associated with severe outcomes; only 1 fracture-containing MDR documented hospitalization. Timing was documented in 12 of the 21 VCF-containing MDRs (57.1%). Among these, 6 occurred within 30 days post-procedure (range: 4-21 days) and 6 occurred beyond 30 days (range: 33-294 days). Timing was not reported in the remaining 9 reports. Given the limited number of reports with timing data and the even distribution between early and delayed events, no consistent temporal pattern can be established from this dataset. Patient age was documented in 18 of the 21 VCF-containing MDRs, ranging from 64 to 92 years with a mean of 79 years. Age was not reported in the remaining 3 reports. Bone health status was explicitly documented in only 2 reports: one noted pre-existing osteopenia and one specifically documented the absence of osteoporosis. Post-fracture treatment with bone anabolic agents (teriparatide or abaloparatide) was mentioned in several narratives, suggesting possible underlying bone fragility in some patients, but osteoporosis status at the time of BVNA could not be systematically determined from the available reports. Where fracture level was specified, it consistently involved a vertebral level that had been treated; however, fracture level was not documented in all reports, and at least one fracture occurred in the setting of a reported post-procedural fall. Among the 21 VCF-containing MDRs, a procedural intervention was documented in 17 reports (81.0%). Kyphoplasty or vertebral augmentation was the most frequently reported treatment, performed or planned in 10 cases (47.6%), followed by vertebroplasty in 5 cases (23.8%). Conservative management was noted in 1 report. Outcome was not documented in the remaining 4 reports.

## 4 Discussion

This analysis of 96 unique medical device reports (MDRs) from the FDA MAUDE database provides a descriptive overview of post-market safety events associated with basivertebral nerve ablation (BVNA; Intracept) from 2018 to 2025. Although the MAUDE database cannot determine incidence or causality, the distribution of adverse event reports can identify areas warranting further investigation and inform the design of prospective studies and registries.

Vertebral compression fracture was the most frequently represented adverse event, occurring in 21.9% (21/96) of reports. Two additional reports described posterior element fractures (2.1%, 2/96) and are reported separately. Other represented events included worsening low back pain (10.4%, 10/96) and worsening radicular symptoms (3.1%, 3/96). While the absolute number of MDRs is modest within an otherwise small dataset, this pattern identifies vertebral fracture as a recurring post-market observation. Among the 12 VCF-containing MDRs with timing data, events were evenly distributed between those occurring within and beyond 30 days post-procedure, and no consistent temporal pattern could be established. Only one fracture-related MDR involved hospitalization.

Prior observational data suggest that vertebral fractures may develop within two months of BVNA, with reported incidence as high as 12% in patients with low bone mineral density (mean T-score −3.0), compared with less than 1% in clinical trials that excluded patients with osteoporosis [11,12]. These findings suggest that pre-existing bone fragility may be an important patient-level factor, though the extent to which it influences post-BVNA outcomes requires further prospective investigation. Our MAUDE-derived results are consistent with this hypothesis, underscoring the need for pre-procedure bone health assessment in patients with risk factors for low bone mineral density and individualized risk-benefit discussion in patients with identified osteopenia or osteoporosis.

Detailed pre-procedural imaging review is essential for minimizing procedural risk. Assessment of pedicle morphology, cortical margins, and vascular anatomy on MRI, supplemented by CT when necessary, can reduce the risk of pedicle breach, inadvertent vascular injury, and thermal spread to adjacent neural structures [[13], [14], [15], [16], [17]]. Pedicle width and angulation should inform approach selection, as extrapedicular trajectories carry higher risk of vascular injury and retroperitoneal hematoma, while medial wall breach risks nerve root irritation or injury [14,15,17,18]. Careful probe positioning remains critical regardless of approach.

No MAUDE reports described spinal cord injury, epidural hematoma, or catastrophic neurologic compromise; however, absence of reports in a passive surveillance database does not imply absence of events in clinical practice, and these complications may be underreported. This is consistent with the anatomic safety margin of intraosseous BVNA, which targets vertebral levels (L3-S1) below the conus medullaris. Radicular pain was identified in 3 of the 96 reports (3.1%), which may correspond to rare complications described in the literature, often due to pedicle breach or thermal spread [4,[19], [20], [21], [22], [23]]; however, subjective neurologic symptoms are likely underreported in passive surveillance datasets. Awareness of pedicle proximity to nerve roots and careful probe positioning remain critical to minimizing neurovascular injury.

Vascular complications, such as retroperitoneal or psoas hematoma, have been documented only in isolated reports, usually resulting from malpositioned trochars rather than ablative energy itself [4,[23], [24], [25], [26]]. Seven cases of retroperitoneal hematoma were observed in our MAUDE dataset, consistent with the rarity of such events in prior studies. No reports of infection were identified in this dataset; however, this finding should be interpreted with caution, as absence of reports in a passive surveillance system does not reliably indicate absence of events in clinical practice.

Although hospitalizations were infrequent (9/96), they emphasize that severe complications, while rare, may occur. In most instances, hospitalization coincided with fracture or unresolved pain rather than device malfunction. Reported adverse events with documented timing occurred between the day of the procedure and approximately 300 days post-BVNA, though timing was inconsistently captured across event types and no conclusions can be drawn regarding the clinical significance of event timing from this dataset.

A total of 83 MDRs were submitted to the FDA by manufacturers, 7 by user facilities, and 6 were voluntary reports. Twenty-two devices were returned for analysis according to the database. This limited reporting structure constrains the ability to conduct independent root-cause assessments or correlate device design with outcomes. Similar patterns of manufacturer-dominated reporting and selective event capture have been observed in other MAUDE-based analyses of interventional pain and neuromodulation devices. A 2019 analysis of dorsal root ganglion stimulation reports identified comparable underreporting of subjective outcomes relative to hardware-related events [27], and a 2025 analysis of peripheral nerve stimulation MDRs found that 77.1% of reports did not specify anatomical location, with event capture heavily weighted toward objective complications [28]. Strengthening independent reporting systems and implementing mandatory registry participation could substantially enhance post-market safety surveillance.

Taken together, this MAUDE-based analysis identifies the types of adverse events most represented in post-market reports for BVNA, without implying that reporting frequency reflects true clinical incidence. Vertebral fracture and persistent pain were the most represented events in this dataset; however, given the well-documented underreporting of subjective symptoms in passive surveillance systems, the relative representation of these events should not be interpreted as a meaningful comparison of their actual clinical frequency. For researchers, these findings highlight the value of prospective registries with standardized AE capture to determine true incidence and identify modifiable risk factors. Continued capture and transparent reporting of real-world safety data remain essential, as rare or delayed adverse events may not be detected in limited-duration clinical trials. As next-generation systems (e.g., the Stryker BVNA platform approved in 2025) enter the market, ongoing post-market surveillance will be critical to determine whether complication profiles differ by device design or patient selection.

### 4.1 Limitations

This study is constrained by the inherent limitations of passive surveillance. The MAUDE database depends primarily on manufacturer-reported data, so underreporting and selective reporting from non-mandated sources can occur. MAUDE does not provide information on the total number of BVNA procedures performed and, therefore, cannot be used to determine the real-world incidence of adverse events. Many narratives lacked clinical context such as patient demographics, bone health status, vertebral level, or timing of events, which limits interpretation. Medication history, including anticoagulant or antiplatelet use, was not systematically captured in MDR narratives and could not be assessed. Because case identification relied on keyword parsing, alternative terminology may have resulted in missed or misclassified events. Objective complications such as fractures may be overrepresented compared with subjective symptoms like pain. Causality cannot be determined, as hospitalizations or deaths may be coincidental rather than directly related to the device or procedure. Moreover, our analysis does not capture complications arising from off-label use of alternative technologies such as tumor ablation systems that some clinicians employ to perform BVNA-like procedures. Finally, the low rate of device returns and the focus on a single system (Intracept) preclude comprehensive root-cause analysis or comparison with newer devices. This analysis is restricted to devices marketed in the United States and reports submitted to the FDA MAUDE database; findings are not generalizable to BVNA practice outside the United States.

## 5 Conclusion

In summary, this MAUDE-based analysis provides a descriptive overview of post-market adverse event reports associated with BVNA. Vertebral fracture was the most represented post-market signal, with variable timing that did not support a consistent delayed pattern. Neurologic, vascular, and infectious complications were infrequently represented, though passive surveillance data likely underestimate their true occurrence. Importantly, no conclusions regarding incidence or causality can be drawn from this dataset. These observations identify priorities for further investigation rather than serving as clinical mandates. Robust prospective studies and registries with standardized follow-up, pre-procedural bone health documentation, and systematic AE capture are required to more precisely define the long-term safety profile of BVNA and to assess whether emerging device platforms alter complication patterns observed to date.

## Funding

Funding for this study was provided by the Mitchell and June Morris Foundation and the Skaggs Foundation for Research.

## Conflict of interest disclosures

Zachary L. McCormick, MD serves on the Board of Directors of the International Pain and Spine Intervention Society (IPSIS), has research grants from Avanos Medical, Boston Scientific, Presidio Medical, Saol Therapeutics, Spine Biopharma, SPR Therapeutics, and Stratus Medical (paid directly to the University of Utah), and also past consultancies with Avanos Medical, Saol Therapeutics, Stryker, and OrthoSon (all relationships ended). Allison Glinka Przybysz, MD has research grants from Medtronic and Avanos Medical (paid directly to the University of Utah). Alexandra E. Fogarty, MD serves on the IPSIS Board of Directors.
